# Retrospective Data Insight into the Global Distribution of Carbapenemase-Producing *Pseudomonas aeruginosa*

**DOI:** 10.3390/antibiotics10050548

**Published:** 2021-05-09

**Authors:** Min-Ge Wang, Zhi-Yong Liu, Xiao-Ping Liao, Ruan-Yang Sun, Run-Bo Li, Yan Liu, Liang-Xing Fang, Jian Sun, Ya-Hong Liu, Rong-Min Zhang

**Affiliations:** 1 National Risk Assessment Laboratory for Antimicrobial Resistance of Animal Original Bacteria, South China Agricultural University, Guangzhou 510642, China; mingewang@stu.scau.edu.cn (M.-G.W.); xpliao@scau.edu.cn (X.-P.L.); sunruanyang123@stu.scau.edu.cn (R.-Y.S.); runboli@stu.scau.edu.cn (R.-B.L.); fanglx@scau.edu.cn (L.-X.F.); jiansun@scau.edu.cn (J.S.); lyh@scau.edu.cn (Y.-H.L.); 2 Laboratory of Veterinary Pharmacology, College of Veterinary Medicine, South China Agricultural University, Guangzhou 510642, China; 3 Department of Emergency Medicine, The Third Affiliated Hospital, Sun Yat-Sen University, Guangzhou 510630, China; lzhyong@mail.sysu.edu.cn; 4 Guangdong Laboratory for Lingnan Modern Agriculture, Guangzhou 510642, China; 5 Tianhe District Wushan Community Health Service Center, Guangzhou 510640, China; zrmzrm@cau.edu.cn

**Keywords:** carbapenemase, *P. aeruginosa*, global distribution, MLST, ARGs

## Abstract

This study aimed to determine the global distribution and molecular characteristics of carbapenemase-producing *Pseudomonas aeruginosa* isolates. A total of 328 (11.1%, 328/2953) carbapenemase-producing *P. aeruginosa* isolates from humans were obtained from public databases as of October 2019. Of which, the *bla*_VIM_ and *bla*_IMP_ genes were the most prevalent carbapenemases in the *P. aeruginosa* isolates. These carbapenemase-producing *P. aeruginosa* isolates possessed 34 distinct sequence types (STs) and six predominated: ST357, ST823, ST308, ST233, ST175 and ST111. The ST357 and ST823 isolates were primarily found detected in Asia and all ST175 isolates were found in Europe. The ST308, ST233 and ST111 isolates were spread worldwide. Further, all ST823 isolates and the majority of ST111, ST233 and ST175 isolates carried *bla*_VIM_ but ST357 isolates primarily carried *bla*_IMP_. ST308 isolates provide a key reservoir for the spread of *bla*_VIM_, *bla*_IMP_ and *bla*_NDM_. WGS analysis revealed that ST111 carried a great diversity of ARG types (n = 23), followed by ST357 (n = 21), ST308 (n = 19), ST233 (n = 18), ST175 (n = 14) and ST823 (n = 10). The ST175 isolates carried a more diversity and frequent of aminoglycoside ARGs, and ST233 isolates harbored more tetracycline ARGs. Our findings revealed that different carbapenem resistance genes were distributed primarily in variant STs of *P. aeruginosa* isolates, these isolates also possessed an extensive geographical distribution that highlights the need for surveillance studies that detect carbapenemase-producing *P. aeruginosa* isolates in humans.

## 1. Introduction

The antimicrobial-resistant ESKAPE pathogens (*Enterococcus faecium*, *Staphylococcus aureus*, *Klebsiella pneumoniae*, *Acinetobacter baumannii*, *Pseudomonas aeruginosa* and *Enterobacter* spp.) are a global threat to human health and include four Gram-negative bacteria [1]. In particular, *K. pneumoniae* ST307 isolates and *Salmonella enterica* serotype Kentucky ST198 from the USA and Egypt, respectively, have now worldwide dissemination [2,3]. *A. baumannii* ST195 isolates are distributed widely in eight countries and isolates recovered from different locations may present less genomic sequence similarity [4]. However, there is paucity of data regarding the global distribution of *P. aeruginosa* isolates.

Carbapenem antibiotics are generally considered last-line agents for the treatment of severe cases of *P. aeruginosa* infections [5]. However, the recent increases in the prevalence of carbapenem-resistant *P. aeruginosa* nosocomial isolates is great concern [5,6]. Evidence suggests that patients who are infected by carbapenem-resistant pathogens have an increased likelihood of morbidity and mortality compared with those infected by susceptible pathogens [7]. The clinical use of carbapenems is currently restricted as a result of the prevalence of carbapenem resistance genes. More seriously, mobile genetic elements have facilitated their rapid dissemination. So far, *P. aeruginosa* isolates producing *bla*_IMP_, *bla*_VIM_, *bla*_NDM_, or *bla*_KPC_ have been detected in various countries [8]. These cases of carbapenemase-producing *P. aeruginosa* are currently localized to a hospital or a country. Therefore, a large-scale survey to detect carbapenemase-producing *P. aeruginosa* worldwide needs to be further explored.

In this study, we employed genomic analysis to investigate the prevalence and global distribution characteristics of carbapenemase-producing *P. aeruginosa* isolates from human and we characterized their molecular characteristics, diversity, antibiotic resistance genes (ARGs) and virulence genotypes of these pathogens.

## 2. Results

### 2.1. Geographical Distribution of the Carbapenemase-Producing P. aeruginosa Identified from the Genome Database

**Figure 1:**
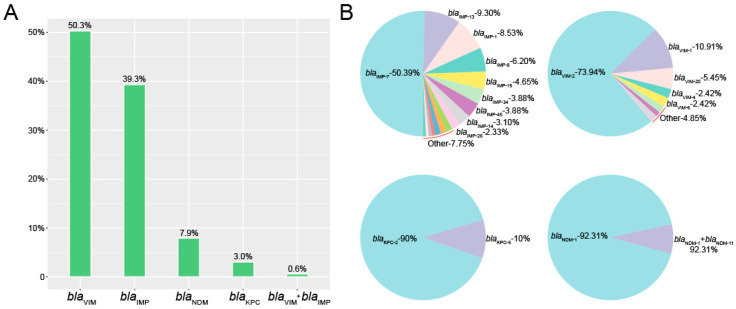
Identification of carbapenemase-producing *P. aeruginosa* isolates. (**A**) The numbers of carbapenem resistance genes in *P. aeruginosa* isolates. (**B**) The rates and numbers of variants in carbapenem resistance genes.

In this study, we first utilized a public database to evaluate the global distribution of carbapenemase-producing *P. aeruginosa* isolates. We identified 2953 *P. aeruginosa* human isolates and 11.1% (328/2953) of the entries indicated the presence of carbapenem resistance genes. Among the carbapenemase-producing *P. aeruginosa* isolates, the most prevalent were *bla*_VIM_ (50.3%, 165/328), *bla*_IMP_ (39.3%, 129/328), *bla*_NDM_ (7.9%, 26/328) and *bla*_KPC_ (3.0%, 10/328). There were no isolates positive for *bla*_OXA-48-like_. Interestingly, two *P. aeruginosa* isolates carried both *bla*_VIM_ and *bla*_IMP_ (Figure 1A).

Within the group of 165 *bla*_VIM_-positive *P. aeruginosa* in this study, *bla*_VIM-2_ accounted for almost 75% of the total, from nine possible variants (73.94%, 122/165). Furthermore, within the 129 members of the *bla*_IMP_ group, *bla*_IMP-7_ represented half of the total (50.39%, 65/129). The *bla*_NDM-1_ and *bla*_KPC-2_ were the most prevalent in *bla*_NDM_- and *bla*_NDM_- positive isolates respectively (Figure 1B).

These carbapenemase-producing *P. aeruginosa* isolates were distributed across 40 countries in Asia (n = 14), Europe (n = 14), Americas (n = 7), Africa (n = 4) and Australia (n = 1). The countries possessing the greatest numbers of carbapenemase-producing *P. aeruginosa* isolates were Indonesia (50.45%, 112/222), India (23.08%, 9/39), Italy (17.72%, 45/254), China (14.81%, 8/54), Germany (11.48%, 7/61), and Spain (11.30%, 20/177) (Table A1). These data demonstrate that Asia has been severely contaminated by carbapenemase-producing *P. aeruginosa* isolates. In the current data, we identified a high incidence of both *bla*_VIM_- and *bla*_IMP_-positive *P. aeruginosa* from Indonesia (*bla*_VIM_: 27.3%, 45/165; *bla*_IMP_: 51.9%, 67/129) and Italy (*bla*_VIM_: 18.8%, 31/165; *bla*_IMP_: 10.1%, 13/129) compared with other countries (Table A2). This suggested that Indonesia and Italy have been severely contaminated by carbapenemase-producing *P. aeruginosa* isolates.

The sample types for our group of 328 carbapenemase-producing *P. aeruginosa* isolates included throat swabs (12.8%, n = 42), urine (11.9%, n = 39), rectal swabs (11.6%, n = 38), blood (11.0%, n = 36), bronchial aspirates (9.8%, n = 32), sputum (8.2%, n = 27) and other sites (10.8%, n = 26), such as wounds, abscesses, cornea, groin and so on, but the source information of the remaining was missing (26.8%, n = 88) (Table A3).

### 2.2. Molecular Characterization of the Carbapenemase-Producing P. aeruginosa

**Figure 2:**
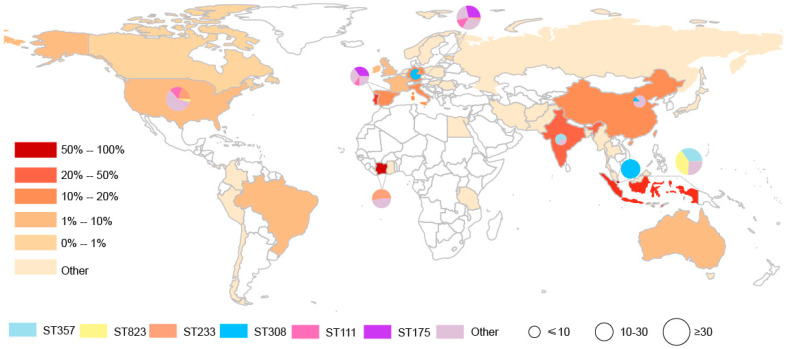
Geographic distribution and MLST diversity of carbapenemase-producing *P. aeruginosa* isolates. The presence of the carbapenemase-producing *P. aeruginosa* isolates is indicated by brown; the pie chart represents MLST diversity.

**Figure 3:**
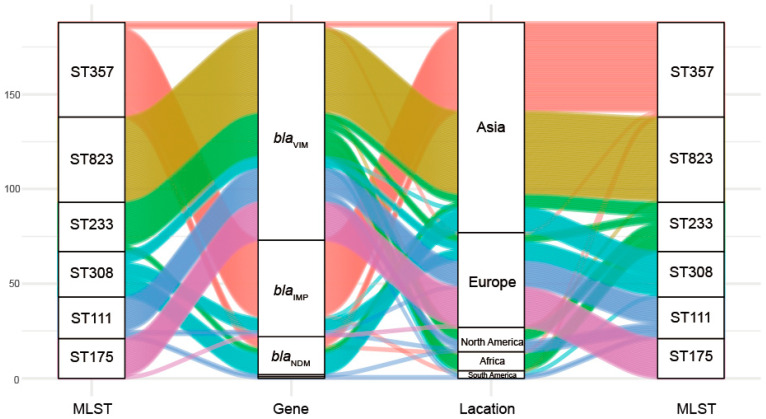
Associations of MLST with antibiotic resistance and geographic locations for the carbapenemase-producing *P. aeruginosa* isolates.

**Figure A1:**
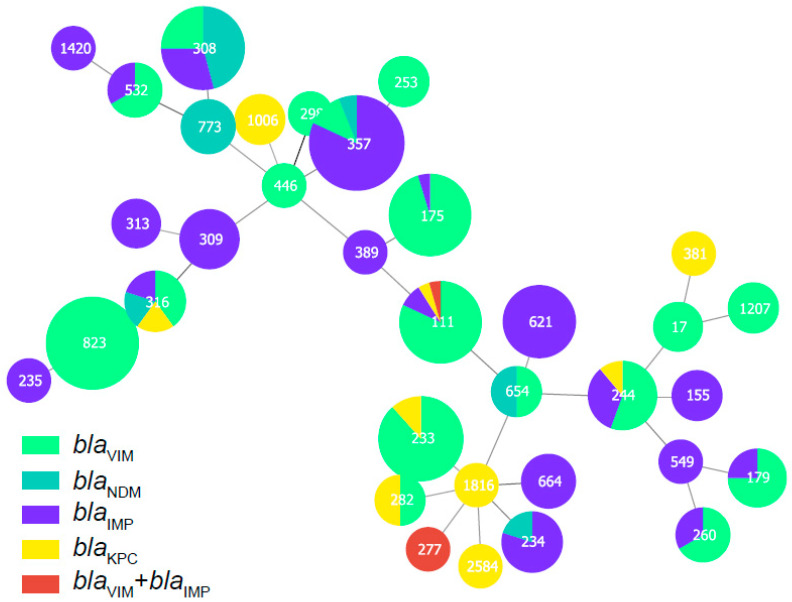
Minimum-spanning tree of carbapenemase-producing *P. aeruginosa* isolates based on a core-genome MLST (cgMLST). Each node within the tree represents a cgMLST type, with diameters scaled to the number of isolates belonging to that type.

This group of 328 carbapenemase-producing *P. aeruginosa* isolates possessed 34 distinct ST and six predominated: ST357 (15.24%, n = 50), ST823 (13.72% n = 45), ST233 (7.93%, n = 26), ST308 (7.32%, n = 24), ST111 (6.71%, n = 22) and ST175 (6.40%, n = 21). Interestingly we could not find a matching ST for 66 of the database isolates (Table A4). Minimum-spanning trees were constructed using 34 distinct STs of 262 carbapenemase-producing *P. aeruginosa* isolates, and the results showed that ST111 might be an ancestral isolate and differentiated into a large number of STs among carbapenemase-producing *P. aeruginosa* isolates (see Appendix A Figure A1). We further explored the clonal relatedness of these isolates in which 10 countries possessed carbapenemase-producing *P. aeruginosa* > 5. The ST types were highly diverse such as for the eleven unique STs in USA, nine for Italy and six for China and Spain, respectively. In contrast, Germany (n = 2), India (n = 1) and Singapore (n = 1) possessed a low level of diverse (Figure 2). More significantly, we identified for the first time that different carbapenem resistance genes were distinctly distributed among the variant STs (Figure 3). All ST823 isolates and the majority of ST111, ST233 and ST175 isolates carried *bla*_VIM_ while the majority of ST357 isolates carried *bla*_IMP_. Furthermore, ST308 isolates provided a reservoir for the spread of *bla*_VIM_, *bla*_IMP_ and *bla*_NDM_ (Figure 3). In addition, there were distinctive geographical distributions for these variant STs. For instance, ST357 and ST823 isolates were primarily from Asia and all ST175 isolates were from Europe. However, ST308, ST233 and ST111 isolates were globally distributed (Figure 3).

### 2.3. Other ARGs

**Figure A2:**
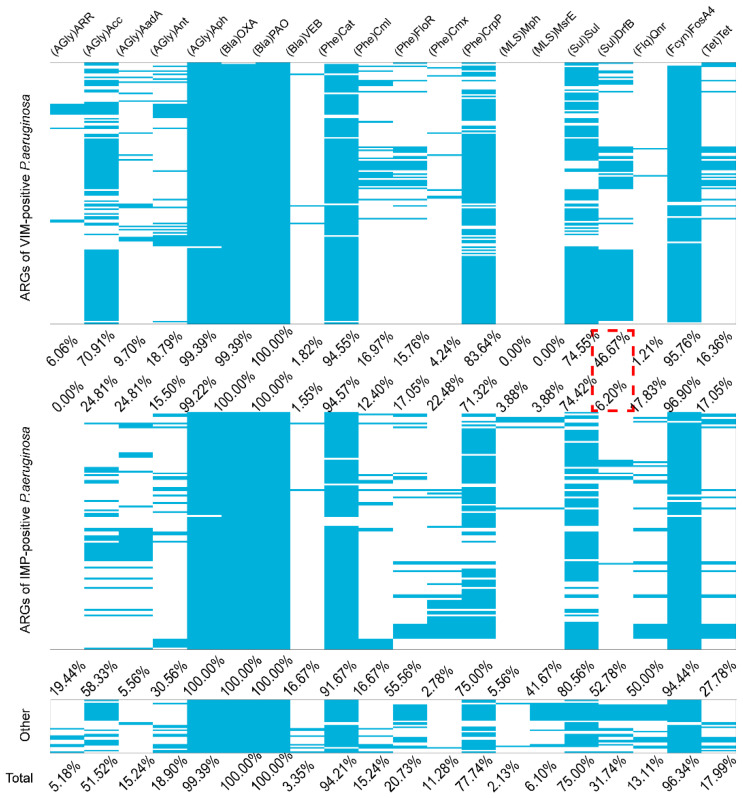
ARGs present among the *bla*_VIM-_ and *bla*_IMP_-positive *P. aeruginosa* isolates found in this study. The blue represents positive for ARGs in the *bla*_VIM-_ and *bla*_IMP_-positive *P. aeruginosa* isolates. The red dotted line represents a special value of worth noting

**Figure A3:**
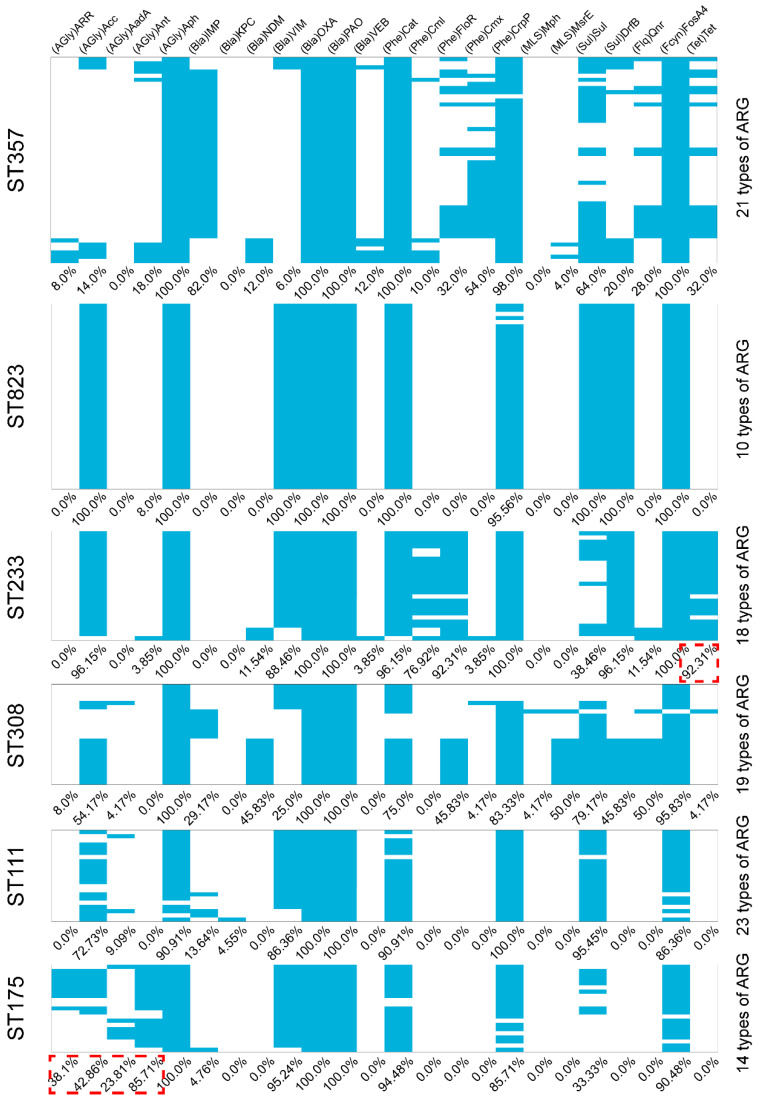
ARGs present among the six most prevalent STs in carbapenemase-producing *P. aeruginosa* isolates. The blue represents positive for ARGs in the carbapenemase-producing *P. aeruginosa* isolates. The red dotted line represents a special value of worth noting

We also identified the presence of other ARGs from the carbapenemase-producing *P. aeruginosa* isolates. Almost all isolates carried *aph*, *bla*_OXA_, *bla*_PAO_, *cat* and *fosA4* that conferred resistance to aminoglycosides, β-lactams, chloramphenicol and fosfomycin. Interestingly, *drfB* that confers resistance to sulfonamides was detected in *bla*_VIM_-positive *P. aeruginosa* at a higher prevalence than in *bla*_IMP_-positive *P. aeruginosa* (Appendix A Figure A2). Generally, the ARGs types among the different STs varied greatly. In particular, the largest numbers of ARGs types were present in ST111 (n = 23), ST357 (n = 21), ST308 (n = 19), ST233(n = 18), ST175 (n = 14) and ST823 (n = 10) (Appendix A Figure A3). The diversity and frequency of aminoglycoside resistance genes carried by ST175 were significantly higher than for the other STs and tetracycline resistance genes carried by ST233 were more numerous than for the other STs (Appendix A Figure A3). These data indicate that the six predominant STs for our group of carbapenemase-producing *P. aeruginosa* isolates possessed shared ARGs and at the same time carried unique ARGs.

### 2.4. Virulence Factor

**Figure A4:**
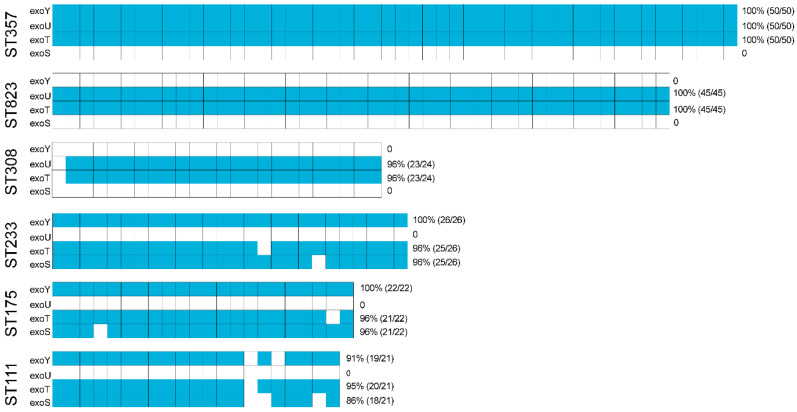
Virulence factors present in carbapenemase-producing *P. aeruginosa* isolates. The blue represents positive for virulence factor in the carbapenemase-producing *P. aeruginosa* isolates.

In our group of carbapenemase-producing *P. aeruginosa* isolates, we detected the presence of *exoY*, *exoU* and *exoT* that coexisted in ST357 isolates, while *exoU* and *exoT* were found together in ST823 and ST308 isolates (Appendix A Figure A4). Interestingly, ST233 isolates possessed the same complement of virulence factors as the ST111 and ST175 isolates (Appendix A Figure A3). These results revealed that the distribution of these cytotoxin genes was not uniform among carbapenemase-producing *P. aeruginosa* isolates, none of the isolates possessed all the cytotoxin genes.

## 3. Discussion

Carbapenems are the most effective antimicrobial agents against serious infections caused by multidrug-resistant Gram-negative bacilli [9]. However, carbapenem-resistant *P. aeruginosa* are emerging worldwide with increasing reports of carbapenemase-producing isolates, such as China, Singapore, Canada, Germany, Spain, US and so on [8,10,11,12,13,14]. In this study, 328 carbapenemase-producing *P. aeruginosa* isolates were collected from public database, and distributed across from 40 countries. Of which, the countries possessing the greatest numbers of carbapenemase-producing *P. aeruginosa* isolates were Indonesia, India, Italy, China, Germany and Spain, further demonstrate that Asia has been severely contaminated by carbapenemase-producing *P. aeruginosa* isolates.

Among the carbapenemase-producing *P. aeruginosa* isolates, the most prevalent were *bla*_VIM_, *bla*_IMP_, *bla*_NDM_ and *bla*_KPC_. The VIM family contains the most important carbapenemases among *P. aeruginosa* strains, while *bla*_NDM_ and *bla*_KPC_ are the most prevalent for *E. coli* and *K. pneumoniae*, respectively [15,16,17]. There is a distinct species preference associated with possession of particular carbapenemase variants. In addition, the high incidence of both *bla*_VIM_- and *bla*_IMP_-positive *P. aeruginosa* from Indonesia and Italy compared with other countries. This is consistent with previous research; Indonesia and Italy have been severely contaminated by carbapenemase-producing *P. aeruginosa* isolates [18,19]. Within the group of 165 *bla*_VIM_-positive *P. aeruginosa* in this study, *bla*_VIM-2_ accounted for almost 75% of the total from nine possible variants. Furthermore, within the 129 members of the *bla*_IMP_ group, *bla*_IMP-7_ represented half of the total. Consistent with previous studies, *bla*_IMP-7_ and *bla*_VIM-2_ were the dominant variants in carbapenemase-producing *P. aeruginosa* isolates from the Czech Republic and Spain, respectively [6,20].

This group of 328 carbapenemase-producing *P. aeruginosa* isolates possessed 34 distinct ST and six predominated: ST357, ST823, ST233, ST308, ST111 and ST175. Core-genome analysis showed that ST111 might be an ancestral isolate and differentiated into a large number of STs among carbapenemase-producing *P. aeruginosa* isolates. This is consistent with previous studies, the international high-risk clone ST111 is the founder of a subgroup from which an important number of STs derive [21]. Interestingly, we identified for the first time that different carbapenem resistance genes were distinctly distributed among the variant STs. All ST823 isolates and the majority of ST111, ST233 and ST175 isolates carried *bla*_VIM_ while the majority of ST357 isolates carried *bla*_IMP_. Furthermore, ST308 isolates provided a reservoir for the spread of *bla*_VIM_, *bla*_IMP_ and *bla*_NDM_. In addition, there were distinctive geographical distributions for these variant STs. For instance, ST357 and ST823 isolates were primarily from Asia and all ST175 isolates were from Europe. However, ST308, ST233 and ST111 isolates were globally distributed. Consistent with these results, ST823 isolates that harbored *bla*_VIM-2_ have been identified in multiple Asian countries [18,22]. ST233 is an internationally recognized high-risk clone and frequently associated with carbapenemase production as well as exhibiting resistance to all antimicrobial drugs, and is present in at least 12 countries [21,23]. Additionally, 90% of multidrug resistant isolates belonged to only three clones and classified as the major international MDR/XDR high-risk clones: ST175, ST111 and ST235 [24]. In particular, the ST111 high-risk clonal isolates have also been found associated with *bla*_VIM_ globally distribution and *bla*_VIM_-positive ST175 isolates have been detected in Germany and Spain. ST357 is also frequently but not exclusively associated with possession of the carbapenemase IMP gene in Asia [6,23]. The *bla*_NDM_, *bla*_VIM_ and *bla*_IMP_ positive ST308 isolates have previously identified in Asian countries [25], we found *bla*_VIM_- and *bla*_IMP-_positive ST308 *P. aeruginosa* isolates in Europe.

We also identified the presence of other ARGs from the carbapenemase-producing *P. aeruginosa* isolates. Almost all isolates carried *aph*, *bla*_OXA_, *bla*_PAO_, *cat* and *fosA4* that conferred resistance to aminoglycosides, β-lactams, chloramphenicol and fosfomycin [23,26]. It is similar to carbapenemase-producing *E. coli* isolates: carbapenem resistance genes often co-existed with other antibiotic resistance genes, conferring resistance to multiple antimicrobials [27,28].

The possession of specific virulence genes by a pathogen including *P. aeruginosa*, is a relevant independent marker of potential disease-causing potential [29]. *P. aeruginosa* possesses numerous virulence factors and one of the most important is its possession of a type III secretion system (TTSS). This system functions to inject effector cytotoxins encoded by *exoS, exoT, exoU* and *exoY* into host cells [24]. In our group of carbapenemase-producing *P. aeruginosa* isolates, we detected the presence of *exoY*, *exoU* and *exoT* that co-existed in ST357 isolates, while *exoU* and *exoT* were found together in ST823 and ST308 isolates. The *exoU* genotype has been associated with increased early mortality and is a potential prognostic biomarker in *P. aeruginosa* infections as an indicator of predicated disease severity [24]. In agreement with this, we found that the ST111 and ST175 high-risk clones possessed *exoY*, *exoT* and *exoS* [6]. Interestingly, ST233 isolates possessed the same complement of virulence factors as the ST111 and ST175 isolates. These results revealed that the distribution of these cytotoxin genes was not uniform among carbapenemase-producing *P. aeruginosa* isolates, none of the isolates possessed all the cytotoxin genes and possession of *exoS* and *exoU* appeared to be mutually exclusive. Our findings are therefore in agreement with previous studies showing that *exoT* and *exoY* were present in the vast majority of strains but *exoS* and *exoU* were nearly mutually exclusive among our *P. aeruginosa* clinical isolates [29].

We acknowledge that our study mainly has two limitations. Firstly, the isolates collection was only dependent on the public repository that could not harbor all the *P. aeruginosa* in the world. Secondly, we could not obtain strains for further research. For instance, the levels of antimicrobial susceptibility and evaluated the transfer of carbapenem resistance genes.

## 4. Materials and Methods

### 4.1. Materials

A total of 5208 assembled genomes and information of *P. aeruginosa* isolates were downloaded from the NCBI database as of October 2019. Human-derived isolates (n = 2953) were filtered according to detailed information from the *P. aeruginosa* database (https://www.ncbi.nlm.nih.gov/pathogens, accessed on 19 October 2019).

### 4.2. Methods

All the *P. aeruginosa* isolates were applied to a filter for the presence of at least one of the five major acquired carbapenem resistance genes (*bla*_KPC_, *bla*_NDM_, *bla*_IMP_, *bla*_OXA-48-like_ and *bla*_VIM_) using ABRicate (https://github.com/tseemann/abricate, accessed on 7 January 2020) The *bla*_KPC_, *bla*_NDM_, *bla*_IMP_, *bla*_OXA-48-like_ and *bla*_VIM_ were the most broadly spread carbapenemases [30]. Multilocus sequence types (MLST) were identified using Github (https://github.com/tseemann/mlst, accessed on 7 January 2020). *P. aeruginosa* isolates virulence factors were identified using the Virulence Factor Database (http://www.mgc.ac.cn/VFs/main.htm, accessed on 7 January 2020). Epidemic ST clones (The STs with the higher detection and distributed in the world) for the carbapenemase-producing *P. aeruginosa* isolates selections were used to analyze associations between STs, geographic location and carbapenem resistance genes. The map was generated using R 3.6.0 using the rworidmap package (https://cran.r-project.org/web/packages/rworldmap/, accessed on 19 July 2020). Minimum-spanning tree of carbapenemase-producing *P. aeruginosa* isolates based on a core-genome MLST was constructed using PHYLOViZ software of BIGSdb [31,32].

## 5. Conclusions

In conclusion, we identified 328 carbapenemase-producing *P. aeruginosa* human clinical isolates from public databases and the carbapenem resistance genes *bla*_VIM_ and *bla*_IMP_ were the most prevalent and present in ~50 and 39% of the isolates, respectively. These strains possessed diverse ST types in most countries and eleven unique STs were identified in the USA, nine in Italy and six in China and Spain, respectively. In contrast, Germany (n = 2), India (n = 1) and Singapore (n = 1) possessed a low level of diverse. We found six prevalent STs and comprised distinct groups. For instance, all ST823 isolates and the majority of ST111, ST233 and ST175 isolates carried *bla*_VIM_, while the majority of ST357 isolates carried *bla*_IMP_. The ST308 isolates provided a reservoir for the spread of *bla*_VIM_, *bla*_IMP_ and *bla*_NDM_. The distribution of the genes encoding these cytotoxins was not uniform among carbapenemase-producing *P. aeruginosa* isolates, none possessed all cytotoxins, and some of them, particularly *exoS* and *exoU*, appeared to be mutually exclusive. These results suggest that surveillance studies for carbapenemase-producing *P. aeruginosa* isolates in humans are urgently needed.

## Data Availability

The data for this manuscript is available from correspondence author.

## Appendix A

**Table A1:** Distribution of carbapenemase-positive *P. aeruginosa* in various countries.

Continents	Countries	Carbapenemase-Positive *P. aeruginosa*	Total of *P. aeruginosa*	Rate
Asia (162)	Indonesia	112	222	50.45%
	China	8	54	14.81%
	Iran	1	1	
	Japan	3	6	
	Afghanistan	2	3	
	India	9	39	23.08%
	Bangladesh	1	1	
	Myanmar	1	1	
	Turkey	2	2	
	Singapore	11	11	
	Malaysia	4	16	
	Lebanon	4	8	
	Thailand	3	25	
	Pakistan	1	1	
Europe (101)	Russia	2	9	
	Netherlands	2	6	
	Italy	45	254	17.72%
	Portugal	9	16	
	Germany	7	61	11.48%
	Norway	2	2	
	Estonia	1	148	0.68%
	France	5	65	7.69%
	Hungary	1	1	
	United Kingdom	3	181	1.66%
	Belgium	1	9	
	Poland	1	2	
	Spain	20	177	11.30%
	Sweden	2	6	
America (40)	Brazil	4	87	4.60%
	Colombia	1	3	
	Peru	2	2	
	Chile	1	2	
	Panama	1	1	
	USA	30	767	3.91%
	Canada	1	497	0.20%
Africa (23)	Ghana	4	5	
	Cote d’Ivoire	17	18	
	Egypt	1	1	
	Tanzania	1	16	
Oceania (2)	Australia	2	117	1.71%

**Table A2:** Distribution of VIM- and IMP-positive *P. aeruginosa* in various countries.

VIM			IMP		
Countries	Numbers	Rate	Countries	Numbers	Rate
Indonesia	45	27.3%	Indonesia	67	51.9%
Italy	31	18.8%	Italy	13	10.1%
USA	19	11.5%	China	7	5.4%
Spain	16	9.7%	cote	6	4.7%
cote	11	6.7%	Germany	6	4.7%
Portugal	11	6.7%	Ghana	4	3.1%
India	5	3.0%	Malaysia	4	3.1%
Lebanon	4	2.4%	Spain	4	3.1%
United Kingdom	3	1.8%	USA	4	3.1%
Afghanistan	2	1.2%	France	3	2.3%
Netherlands	2	1.2%	Australia	2	1.6%
Norway	2	1.2%	Peru	2	1.6%
Russia	2	1.2%	Thailand	2	1.6%
Brazil	1	0.6%	Iran	1	0.8%
Chile	1	0.6%	Sweden	1	0.8%
China	1	0.6%	Tokyo	1	0.8%
Colombia	1	0.6%	Turkey	1	0.8%
Egypt	1	0.6%	Belgium	1	0.8%
France	1	0.6%			
Germany	1	0.6%			
Panama	1	0.6%			
Peru	1	0.6%			
Sweden	1	0.6%			
Turkey	1	0.6%			
Bangladesh	1	0.6%			

**Table A3:** The origin of the carbapenemase-positive *P. aeruginosa* isolates.

Body Site	Number of Isolates	Rate
Throat	42	17.50%
Urine	39	16.25%
Rectal	38	15.83%
Blood	36	15.00%
Bronchial	32	13.33%
Sputum	27	11.25%
Wound	11	4.58%
Abscess	4	1.67%
Cornea	2	0.83%
Feces	2	0.83%
Lung	2	0.83%
Eye	1	0.42%
Groin	1	0.42%
Peritoneal fluid	1	0.42%
Pleural fluid	1	0.42%
Knee	1	0.42%
Unknown	88	26.83%

**Table A4:** The number of carbapenemase-positive *P. aeruginosa* of various MLST types.

MLST	Number of Carbapenemase-Positive *P. aeruginosa*
Unknown	66
ST357	50
ST823	45
ST233	26
ST308	24
ST111	22
ST175	21
ST621	12
ST244	9
ST234	5
ST316	5
ST179	4
ST260	3
ST309	3
ST532	3
ST664	3
ST773	3
ST155	2
ST253	2
ST282	2
ST654	2
ST1207	2
ST1006	2
ST17	1
ST235	1
ST277	1
ST298	1
ST313	1
ST381	1
ST389	1
ST446	1
ST549	1
ST1420	1
ST1816	1
ST2584	1
